# 

**Published:** 1891-03

**Authors:** 


### CONTENTS.

### COMMUNICATIONS.

Aneurismal Tumor ........ 109
Salivary Calculous ........ 114
What circumstances and conditions modify the action of Solvents? .. 116
The Health of the Dentists ........ 119
A Temperance Proverb ........ 123
Evolution of Dentistry ........ 124

### SELECTIONS.

Study of Comparative Anatomy ........ 129
Dr. Koch ........ 138
The Composition of Koch's Lymph ........ 139
The New Hypnotic—Somnal ........ 141
The Products of Pathogenic Bacteria ........ 142
Deafness for High Notes ........ 143
The Curve of Health ........ 144
Treatment of Obesity ........ 145
The Value of Time ........ 146
Therapeutic Application of Nitrous Oxide ........ 147
Death from Cocaine Injections ........ 149
A Deciduous Man ........ 150
A Laryngological Curiosity ........ 151
Surgical Shock ........ 151
Tea Drinking and Cold Feet ........ 152
Nasal Obstructions and Spinal Deformities ........ 153
Micro—Organisms in Clothing ........ 153
Hot Water Remedies ........ 154
Sanity and Insanity ........ 154

### EDITORIAL.

Requirements for Admission to University of Michigan Dental Department ........ 155
Scheme of Study for the College of Dental Surgery, University of Michigan ........ 156
Mississippi Valley Dental Association ........ 158
The World's Columbian Dental Meeting, 1893 ........ 158
Whales and Bacteria ........ 159
Bibliographical ........ 160

